# Functional–Structural Plasticity Associated With the Duration of Sports Participation in Lower Limb Amputees: A Multimodal Neuroimaging Study

**DOI:** 10.1155/np/8819722

**Published:** 2026-04-24

**Authors:** Nobuaki Mizuguchi, Tomoya Nakanishi, Shohei Tsuchimoto, Kimitaka Nakazawa

**Affiliations:** ^1^ Department of Life Sciences, Graduate School of Arts and Sciences, The University of Tokyo, Meguro-ku, Tokyo, Japan, u-tokyo.ac.jp; ^2^ Juntendo Administration for Sports, Health and Medical Sciences, Juntendo University, Inzai, Chiba, Japan, juntendo.ac.jp; ^3^ NTT Data Institute of Management Consulting, Inc., Chiyoda-ku, Tokyo, Japan; ^4^ Department of System Neuroscience, National Institute for Physiological Sciences, Nishigonaka Myodaiji, Okazaki, Aichi, Japan, nips.ac.jp

**Keywords:** amputation, diffusion-weighted imaging, fixel-based analysis, reorganization, sports

## Abstract

Structural and functional brain reorganization can occur after long‐term physical activity and lower limb amputation (LLA). A previous study suggested that activation of the primary motor cortex (M1) ipsilateral to the amputated leg during rectus femoris contraction is associated with the amount of sports participation in individuals with LLA. However, the structural basis of ipsilateral M1 activation remains unclear. The aim of this study was to investigate whether ipsilateral M1 activation is associated with white matter microstructure in descending motor pathways. We hypothesized that ipsilateral M1 activation would be related to microstructural properties of the corticoreticular tract (CRT) rather than the corticospinal tract (CST). Twenty‐three individuals with LLA who had participated in sports for varying durations underwent functional magnetic resonance imaging (fMRI) and diffusion‐weighted imaging. During fMRI, percent signal change (PSC) during rectus femoris contraction in the amputated leg was quantified. White matter microstructure was assessed using fixel‐based analysis (FBA). Ipsilateral M1 PSC during contraction of the amputated leg was positively correlated with fiber cross‐section (FC) in the CRT within the same hemisphere, whereas no significant correlation was observed for the CST. No significant correlations were found in control analyses using PSCs during contraction of the rectus femoris in the nonamputated leg, during motor imagery of ankle movements of the amputated leg, or in the visual cortex. These findings indicate a task‐ and hemisphere‐specific functional–structural association between ipsilateral M1 activation and the CRT. Although FC in the CRT was not directly correlated with the amount of sports participation, it may constrain the extent of ipsilateral M1 functional reorganization associated with long‐term sports involvement. Our results highlight a potential role of the CRT, in addition to the CST, in motor plasticity and rehabilitation in individuals with LLA.

## 1. Introduction

Brain function and structure undergo reorganization due to physical activity [1]. This phenomenon encompasses the enlargement of the primary motor cortex (M1) representation associated with a trained limb [2, 3]. In addition, neuroimaging studies suggest that structural changes in M1 are induced by physical activity [4, 5]. Brain function and structure show notable adaptations in individuals with extensive training as brain function and structure were different between experts and healthy controls [6–8]. These observations highlight the importance of investigating the long‐term effects of physical activity on brain reorganization.

Functional and structural reorganization of the brain also occurs after lower limb amputation (LLA) in various brain regions including the primary sensorimotor cortex (SM1). For instance, the enlargement of the representation in M1 contralateral to the amputated limb was observed even after 15 years of amputation [9]. In addition, resting‐state functional connectivity between contralateral M1 and subcortical regions gradually increased in association with time since amputation [10]. The M1 ipsilateral to the amputated limb was also reorganized. For instance, a previous study using transcranial magnetic stimulation (TMS) demonstrated that lateral displacement of the center of gravity of motor output map was observed in both contralateral and ipsilateral M1 in individuals with LLA [11]. In addition, structural changes in corpus callosum (CC) occur limb amputation [9, 12, 13].

A recent case study explored a Paralympic gold medalist with a unilateral LLA who is also the world record holder in the long jump for his category [14]. Notably, this previous study reported the activation of the ipsilateral M1 to the amputated leg during contraction of the rectus femoris in the amputated side. However, this was not evident in the nonamputated leg, in nonathletes with a unilateral LLA, or in able‐bodied long jumpers. Therefore, intensive prosthesis use in the context of sports participation—characterized by high frequency and intensity, as well as challenging balance demands—likely differs from typical daily prosthesis use in driving neural reorganization [14]. Moreover, in a cross‐sectional study, we demonstrated that the degree of ipsilateral M1 activation during contraction of the rectus femoris in the amputated leg was correlated with long‐term sports participation [15]. Thus, ipsilateral M1 activation might be associated with fine motor control of the prosthesis, as it is operated by the femoral muscle and knee joint [16]. It was hypothesized from these studies that the ipsilateral M1 activation would result from long‐term specific use of the prosthesis that is inevitably required in sports activities. In other words, the observed brain reorganization in individuals with LLA with long‐term sports participation likely occurs in a use‐dependent and impairment‐specific manner [17]. However, the structural basis of ipsilateral M1 activation associated with sports participation remains unclear.

In the present study, we focused on functionally essential descending pathways, the corticospinal tract (CST) and corticoreticular tract (CRT), in order to explore the association with the ipsilateral M1 activation observed in individuals with LLA. It is well known that the large part of M1 is directly connected to spinal motoneurons via CST), which is relevant to voluntary control, especially fine motor control of limb muscles. The CRT originates from the M1, premotor cortex, and supplementary motor area and is mainly involved in postural and gait control [18]. To the best of our knowledge, no study has investigated the relationship between ipsilateral M1 activation and motor‐related descending pathways (i.e., the CST and CRT) in individuals with LLA.

To clarify the specific structural pathway associated with ipsilateral M1 activation during contraction of the rectus femoris in the amputated leg, we analyzed diffusion‐weighted images (DWI), evaluated fiber density (FD), and fiber cross‐section (FC) using fixel‐based analysis (FBA) [19]. We created regions of interest (ROIs) of the CST and CRT. As the two M1s are interconnected via the CC [20], the CC is a candidate tract underlying ipsilateral M1 activation. Therefore, we also created the ROI of the CC. We then investigated if the ipsilateral M1 activation, corresponding to the contracted leg during muscle contraction in the amputated leg, would be correlated with FD and FC within these three ROIs. We hypothesized that ipsilateral M1 activation would be associated with the microstructure in the descending motor‐related pathways.

## 2. Experimental Procedures

### 2.1. Participants

Twenty‐three individuals with LLA participated in the present study. There were 17 men and six women aged 42 ± 16 years (mean ± standard deviation) (range: 15–70) who were 20 ± 17 years (range: 1–60) postinjury and had 11 ± 12 years (range: 0–52) of para‐sports experience. All participating individuals with LLA were also enrolled in a previous task‐functional magnetic resonance imaging (MRI) study [15]. Ten participants had received transtibial amputations, and 13 had received transfemoral amputations (10 on the left side). As the previous study suggested that a trend of reorganization seems to be similar between transfemoral and transtibial limb amputees [15], we analyzed data regardless of the location of amputation. The mechanisms of injury were traumatic accidents (*n* = 17), tumors (*n* = 5), and arterial obstruction (*n* = 1). The following eligibility criteria were used: (1) chronic phase after unilateral LLA; (2) no phantom pain or abnormal peripheral sensations in the amputated limbs; (3) absence of medical or neurological diseases; and (4) absence of neurotrauma, psychotropic medications, and MRI contraindications. A previous study suggested that phantom limb pain might affect cortical reorganization in addition to amputation without phantom limb pain [21]. To improve interpretability, we excluded the factor of phantom limb pain.

This study was approved by the Human Ethics Committee at the Graduate School of Arts and Sciences, University of Tokyo (2018, approval number 581‐2) and was conducted in accordance with the Declaration of Helsinki (2000 revision). Prior to the experiment, each participant received a detailed explanation of the experimental procedures and signed a consent form. This study was conducted as part of the Doctoral Dissertation of one of the authors [22].

### 2.2. Experimental Procedures

Prior to MRI measurement, the participants completed several questionnaires, which collected information regarding the following aspects: (1) medical history (all previous diseases); (2) injury date (date of amputation and postamputation duration); and (3) sports history (sports played by the participants at least once a week and the number of years spent playing). The participants completed the scans for functional MRI, T1‐weighted images, and DWI. In the present study, we extracted the individual values of ipsilateral M1 activity during voluntary contractions from the functional MRI data and performed correlation analysis with DWI data (see below). The details of the functional MRI procedure have been previously described [15]. In brief, all participants performed six rhythmic isometric unilateral muscle contraction tasks involving the ankle, knee, and hip joints. The six movements were randomly ordered to impede the prediction of the subsequent task. Both task‐ and rest‐periods lasted 20 s; therefore, each session lasted 268 s including an 8‐s pretrial dummy scan. The participants conducted three sessions. As activity in the rectus femoris muscle in the amputated leg in transfemoral limb amputees could be observed by electromyography (EMG) (Trigno Wireless System; DELSYS, Boston, MA, USA), all participants including transfemoral limb amputees executed the rectus femoris muscle contraction task. For ankle movement with the amputated leg, participants were instructed to imagine a cyclic dorsiflexion/plantarflexion movement using the first‐person perspective. In the present study, we used only the brain activation values during knee muscle contraction because ipsilateral M1 activation was observed during knee muscle contraction using the amputated leg [14, 15]. All participants were instructed to perform voluntary isometric contractions of the knee extensors at a constant rate of 1 Hz following the blinking yellow fixation point on the monitor. Prior to entering the MRI scanner, the participants were allowed to practice contracting each muscle at 20% of their maximum voluntary contraction, ensuring the isolation of muscle activation. EMG signals were digitally converted using an A/D converter system with a sampling rate of 1 kHz (PowerLab System, AD Instruments, Sydney, Australia) and were band‐pass filtered (20–450 Hz). The practice session was terminated when the contractions were isolated from the primary muscles, and 20 consecutive contractions at 20% maximum voluntary contraction were completed without supervision.

### 2.3. Scanning Parameters

All MRI data were acquired using a 3.0‐T MRI scanner with a 64‐channel head coil (MAGNETOM Prisma, Siemens, Germany).

We obtained T2 ^∗^ weighted echo‐planar images reflecting the blood oxygenation level‐dependent signal using the following parameters: repetition time (TR) = 2000 ms, echo time (TE) = 25 ms, flip angle = 90°, field of view (FOV) = 192 mm, 39 contiguous axial slices acquired in an interleaved order, thickness = 3.0 mm, in‐plane resolution = 3.0 mm × 3.0 mm, and bandwidth = 1776 Hz/pixel.

High‐resolution T1‐weighted structural images were acquired using a 3D magnetization‐prepared rapid acquisition with a gradient echo pulse sequence using the following parameters: TR = 2000 ms, TE = 2.9 ms, flip angle = 9.0°, FOV = 256 mm, 176 contiguous axial slices, thickness = 1.0 mm, and in‐plane resolution = 1.0 mm × 1.0 mm.

Whole‐brain DWI was acquired using a double spin‐echo sequence (64 directions; *b*‐value = 2000 s/mm^2^, 84 slices, FOV = 230 mm, voxel size: 1.8 mm isotropic, TR = 3,300 ms, TE = 70 ms, flip angle = 90°). At the beginning of the sequence, one volume without diffusion weighting (*b* = 0 s/mm^2^) was acquired. The acquisition time for the diffusion scan was ~10 min.

### 2.4. Extraction of the Signal Intensity From Functional MRI for Correlation Analysis

For the correlation analysis between M1 activity during the motor task and fixels in motor‐related pathways, we extracted time‐series data of the blood oxygenation level‐dependent signal from functional ROIs in each participant. Subsequently, we calculated the percent signal changes (PSCs) in knee contraction periods relative to the rest periods. The functional ROIs were set at the M1. The coordinates of each functional ROI were defined as the most activated voxels (peak voxels) within each region in the individual analyses using the SPM12 software (v.7771, Wellcome Trust Center for Neuroimaging, University College London, London, UK, https://www.fil.ion.ucl.ac.uk/spm/software/spm12/) rather than anatomical ROIs. We selected this method to capture activation patterns in each individual as postamputation neural reorganization may spatially shift motor representation [11].

After standard preprocessing (realignment, coregistration, normalization, and smoothing), a general linear model was performed at the individual level (i.e., first‐level analysis). We then identified, for each participant, the peak coordinates of the most strongly activated voxel within the right M1. To minimize contamination from the contralateral M1 and noncortical tissue, peak coordinates were searched only within voxels with *x* > 8 and *z* > 70 in MNI space. Functional ROIs were defined as 10‐mm‐diameter spheres using the MarsBaR toolbox (MRC Cognition and Brain Sciences Unit, Cambridge, UK). To extract PSCs, we additionally estimated a separate first‐level GLM using unsmoothed images with the same design matrix as in the main analysis. This approach was used to avoid signal spread across the midline due to spatial smoothing, which could contaminate ipsilateral M1 with activity from the contralateral M1. As control regions, PSCs were also extracted from the contralateral M1 during right knee contractions. In addition, we extracted PSCs from the ipsilateral and contralateral M1 during knee contractions in the nonamputated leg, as well as from the ipsilateral M1 during motor imagery of ankle movements of the amputated leg. For the contralateral M1 during knee contractions in the nonamputated leg and for the ipsilateral M1 during motor imagery of ankle movements of the amputated leg, we used two ROI definitions: (i) the condition‐specific individually determined peak, or (ii) the individual peak coordinate identified from the right knee contraction.

### 2.5. FBA

We performed a standard analysis pipeline for the FBA using MRtrix3 (v.3.0.2, https://www.mrtrix.org/) [19]. First, DWI from participants with left‐side amputations was flipped (left‐to‐right transformation on the *x*‐axis) to place all amputated sides to the right [23, 24]. For preprocessing, denoising, unringing, motion, distortion, and bias field corrections were performed using the *dwidenoise*, *mrdegibbs*, *dwifslpreproc*, and *dwibiascorrect* functions [19, 25, 26]. The group‐averaged white‐matter response was then calculated and used for subsequent analyses. The DWI data were upsampled to a voxel size of 1.25 mm (isotropic) to improve spatial resolution. The fiber orientation distribution (FOD) function was computed using constrained spherical deconvolution, and a maximum spherical harmonic order of 8 was used for calculations [27]. Then, global intensity normalization was conducted for all participants using groupwise registration, and the median *b* = 0 white‐matter value was used as a reference [28]. All FODs were registered to the FOD template to obtain spatial correspondence. The FODs in the study‐specific template space were then segmented to estimate FD, FC, and FDC (i.e., a combined index of FD and FC). FC was log‐transformed before statistical analysis. Finally, the fixel data were smoothed based on the sparse fixel–fixel connectivity matrix using the *fixelfilter* function.

**Figure 1:**
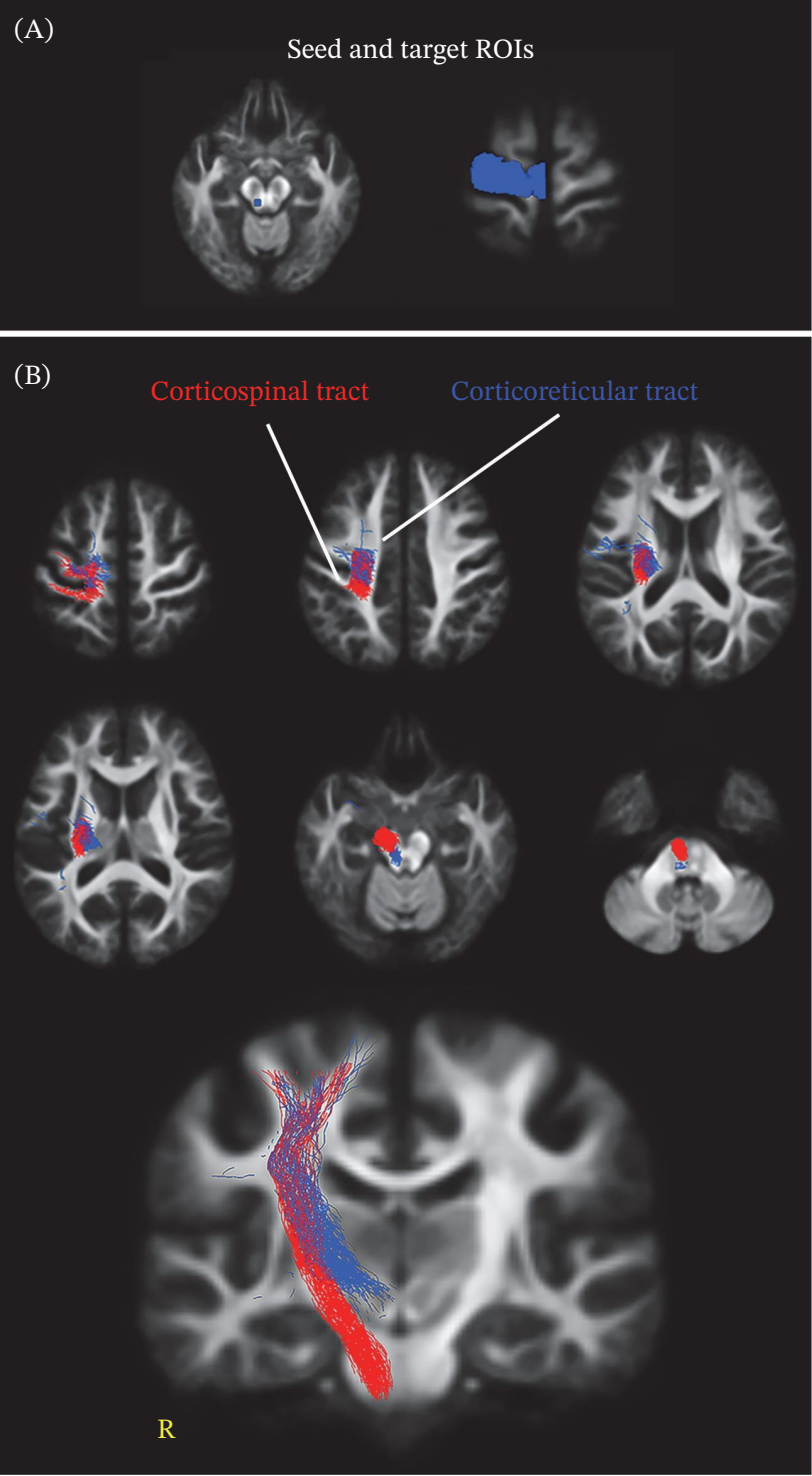
(A) The seed and target regions of interest for creating the corticoreticular tract. (B) Created tractographies of the corticospinal tract and the corticoreticular tract. Note that images of participants with left‐side amputations were flipped to place all amputated sides to the right. ROI, region of interest.

We created fixel masks (i.e., ROIs) for the CC, CST, and CRT. The fixel mask of the CC was created using the ICBM‐DTI‐81 white‐matter labels atlas in FSL (v.6.0.3, https://fsl.fmrib.ox.ac.uk/fsl/fslwiki/). First, the voxel ROI of the CC was transformed into the study‐specific template space using *mrregister* and *mrtransform*. Then, a fixel mask of the CC was created with *voxel2fixel* using the transformed voxel ROI of the CC and the whole‐brain fixel mask. The fixel mask of the CST was generated using the AutoPtx plugin (https://fsl.fmrib.ox.ac.uk/fsl/fslwiki/AutoPtx). First, seed‐ and target‐ROIs for the CST in the FMRIB‐58 1 mm space were transformed to the study‐specific template space using *mrregister* and *mrtransform*. The *tckgen* command was then used to create a tractography of the CST (max length = 250 mm, min length = 10 mm, cutoff value = 0.07, maximum angle = 22.5°, 500 streamlines). For the fixel mask of the CRT, with reference to a previous study [29], the seed‐ and target‐ROIs were set at the manually delineated midbrain tegmentum and precentral gyrus in the Harvard–Oxford atlas, which was transformed to the study‐specific template (Figure 1A). The *tckgen* command was then used to create a tractography of the CRT (max length = 250 mm, min length = 10 mm, cutoff value = 0.07, maximum angle = 22.5°, 500 streamlines). To reduce bias, the tractographies of the CST and CRT were reduced to 300 streamlines using the *tcksift* command. From a visual inspection, the CRT trajectory seemed comparable to that of previous studies [29, 30]. In addition, the CST and CRT were dissociated, at least below the CC (Figure 1A,B). Furthermore, we verified that the statistical results were similar if we used different fixel masks created by 100 or 500 streamlines of tractographies (data not shown). Therefore, the effect of the number of streamlines in the fixel masks was minimal. Subsequently, a combined fixel mask for the right hemisphere was created. That is, the fixel mask included the CC, right CST, and right CRT (i.e., the right fixel mask). For control analyses, the fixel mask for the left hemisphere was also created including the CC, left CST, and left CRT (i.e., the left fixel mask).

Correlation analyses using PSCs of the ipsilateral M1 during amputated knee contraction were performed using the right fixel mask. Age, sex, and years since injury were used as covariates of no interest. Statistical analyses were performed using connectivity‐based fixel enhancement with 5000 permutations by the *fixelcfestats* function [31]. As a control analysis, the correlations using (1) PSCs of the contralateral M1 during knee contraction in the amputated leg; (2) PSCs of the ipsilateral M1 during knee contraction in the nonamputated leg; (3) PSCs of the contralateral M1 during knee contraction in the nonamputated leg; and (4) PSCs of ipsilateral M1 during motor imagery of ankle movement in the amputated leg were also calculated with nuisance regressors of age, sex, and years since injury. In addition, correlation analysis using the duration of sports activity was performed. The statistical threshold was set at *p*  < 0.05 after family‐wise error (FWE) correction for multiple comparisons.

To check whether significant correlation was influenced by outliers, we depicted individual fixel values within significant regions if significant correlation was observed. We created a fixel mask of a significant region and extracted the mean values within the fixel mask using *mrstats* function, individually.

Portions of the manuscript text were revised for clarity and language using ChatGPT. The tool was used only for editing and polishing wording and did not generate or alter any scientific content, analyses, results, or conclusions.

## 3. Results

### 3.1. Correlations Between Activation of the Ipsilateral M1 and Fixels

**Figure 2:**
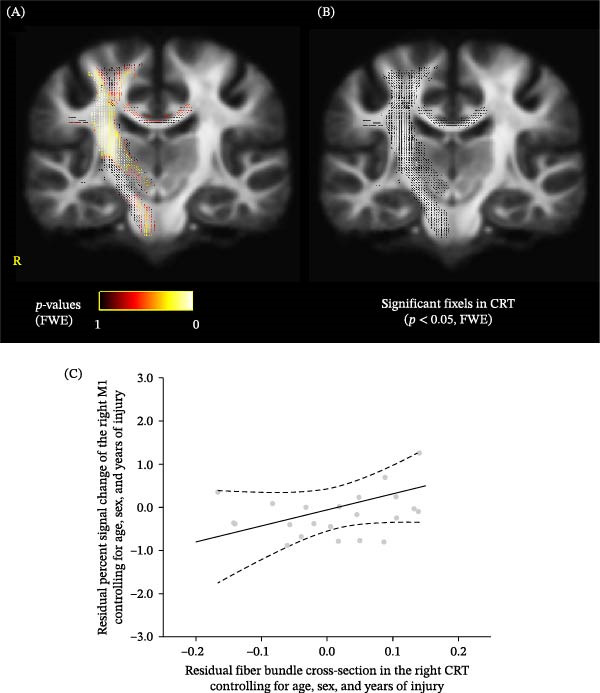
(A) *p*‐Values of fixels for fiber bundle cross‐sections associated with activation in the ipsilateral M1 to the contracted leg during isometric contractions of the rectus femoris in the amputated leg. (B) The fixel mask of the CC, the CST, and the CRT is in black, showing significant fixels. The statistical threshold was set at *p*  < 0.05 after a family‐wise error (FWE) correction for multiple comparisons. (C) Scatter plot of the partial correlation between fiber cross‐sections and the activation in the ipsilateral M1, controlling for age, sex, and years since injury. Solid and dotted lines represent the estimated linear regression and 95% CI, respectively. M1, primary motor cortex; CRT, corticoreticular tract.

The values for the individual PSCs are summarized in Table 1. Correlation analyses demonstrated that FC in the right CRT positively correlated with the PSCs of the ipsilateral M1 during knee contraction in the amputated leg (FWE‐corrected *p*  < 0.05) (Figure 2A,B). A significant correlation was also observed for FC in the descending pathway; however, distinguishing the CRT from the CST is challenging because these tracts partially overlap due to limitations in tract definition (Figure 2A). This correlation was not observed in the CC. To additionally check the relationship between individual FC in the CRT and M1 activity, we plotted residualized values after controlling for age, sex, and years since injury (Figure 2C). The negative residuals in Figure 2C indicate values lower than those predicted based on age, sex, and years since injury. To confirm that the same trend was present without adjustment for these factors, we also plotted the unadjusted values and observed a similar positive trend (Figure S1). These results suggested that functional activity in the ipsilateral M1 was associated with FC in the CRT in the same hemisphere. FD and FDC in the CRT, CST, and CC were not significantly associated with PSCs in the ipsilateral M1 (all FWE‐corrected *p* values > 0.05).

**Table 1:** Values of individual percent signal change during motor task.

Sex	Amputation at tibia/femur	Age (years)	Time since amputation (years)	Time spent sports (years)	Ipsilateral M1 (amputated knee task)	Contralateral M1 (amputated knee task)	Ipsilateral M1 (nonamputated knee task)	Contralateral M1 (nonamputated knee task)	Ipsilateral M1 (amputated ankle task)
M	TT	21	16	6	1.85	3.09	0.01	0.86	0.91
F	TF	20	19	15	0.40	1.16	−0.17	0.30	0.69
M	TF	46	1	1	0.71	0.61	0.05	0.80	0.61
M	TT	32	6	5	0.33	2.07	−0.97	1.31	0.89
M	TF	54	29	20	0.24	1.29	0.72	1.02	−0.10
M	TF	66	49	0	2.07	1.81	0.16	3.00	0.42
M	TT	49	28	10	1.28	0.61	−0.18	3.67	0.80
M	TF	61	54	52	1.35	3.68	0.26	2.22	2.56
M	TT	49	17	15	0.22	0.65	−0.38	0.38	4.35
M	TF	38	18	10	0.39	1.10	0.27	0.99	0.02
M	TF	45	6	6	4.48	1.84	−0.53	5.73	0.19
F	TT	70	60	30	2.05	1.17	−0.25	1.69	3.63
M	TF	69	3	1	0.83	3.33	0.74	2.25	1.64
F	TT	15	1	1	0.89	1.11	0.63	1.10	1.65
M	TF	26	23	13	0.59	1.71	0.12	1.90	0.32
M	TF	46	20	4	0.18	0.79	−0.08	0.42	1.11
M	TF	30	10	6	0.66	1.29	−1.10	1.13	−0.27
M	TT	24	7	3	0.35	2.60	0.47	1.42	0.20
F	TT	53	6	3	0.85	0.43	0.14	0.55	0.30
M	TF	39	23	10	0.20	1.13	−0.18	0.87	1.33
M	TF	37	13	7	0.00	1.60	0.29	1.25	−0.72
F	TT	23	14	10	0.91	1.38	−0.01	2.08	−0.06
F	TF	44	39	20	1.23	1.54	0.24	1.01	0.62
	Mean	42	20	11	0.96	1.56	0.01	1.56	0.92
	SD	16	17	12	0.97	0.88	0.47	1.24	1.21

Abbreviations: M1, primary motor cortex; SD, standard deviation; TF, transfemoral amputations; TT, transtibial amputations.

### 3.2. Results of Control Analyses

No significant results were observed in any control analysis. Specifically, PSCs during knee contractions in the nonamputated leg were not correlated with FC, FD, or FDC in either the contralateral or ipsilateral M1 (all FWE‐corrected *p v*alues > 0.05), regardless of whether PSCs were extracted using the condition‐specific peak or the peak coordinate identified from the right knee contraction. In addition, FC, FD, and FDC were not correlated with ipsilateral M1 PSCs during motor imagery of ankle movements of the amputated leg (all FWE‐corrected *p* values > 0.05). Thus, FC in the right CRT was correlated only with ipsilateral M1 PSCs during knee contractions in the amputated leg, but not with contralateral M1 PSCs during knee contractions in the nonamputated leg or with ipsilateral M1 PSCs during motor imagery of ankle movements of the amputated leg. Moreover, FC, FD, and FDC in the contralateral hemisphere were not correlated with contralateral M1 PSCs during knee contraction in the amputated leg (all FWE‐corrected *p* values > 0.05), indicating that the significant relationship observed in the FC of the CRT was hemisphere‐specific. As a supplementary control analysis, PSCs were extracted from an ROI in the primary visual cortex (V1); the results are provided in the Supporting Information (Table S1). Finally, the duration of sports activity was not correlated with FC, FD, and FDC (all FWE‐corrected *p* values > 0.05).

## 4. Discussion

We tested whether increased ipsilateral M1 activation during unilateral leg muscle contraction in amputated legs was associated with structural properties of motor‐related pathways (i.e., the CST, CRT, and CC). In the tract‐specific FBA, ipsilateral M1 activation during knee contraction in the amputated leg was significantly correlated with FC in the specific portions of right CRT in the same hemisphere. Although the number of significant fixels was small, our results suggest that the trend toward a correlation was more apparent for the CRT than for the CST. This finding is consistent with previous studies suggesting that brain function and structure are coupled [32, 33]. However, no significant relationship was found in the control analysis using the activation in different brain regions. That is, the relationship between the FC in the CRT and M1 activation during knee contraction in the amputated leg was hemisphere‐specific. Moreover, the control analysis also demonstrated that the FC in the right CRT was associated with knee contraction in the amputated leg but not with that in the nonamputated leg. Therefore, the CRT likely did not function to control the healthy leg. In the previous study, ipsilateral M1 activation of the contracted leg during knee contraction in the amputated leg was significantly correlated with prolonged sports participation [15]. However, the duration of sports participation was not correlated with the FC in the CRT. As a limitation of the previous and present studies, the duration of sports participation was assessed but not intensity and frequency of sports participation. Consequently, it is possible that some amputees engaged in sports with high intensity and frequency despite having only a brief history of participation. Therefore, interpreting the function of the increased ipsilateral M1 activation is challenging because longer sports participation does not necessarily equate to more motor training or enhanced motor performance. Although the present study cannot mention the causality, we assumed that two possibilities: (1) long‐term sports participation induced structural changes in motor‐related pathways in association with ipsilateral activation, and (2) amputees with greater FC in the CRT have a higher potential to use ipsilateral M1 and long‐term sports participation would effectively enhance ipsilateral M1 activation for them. Therefore, using a longitudinal experiment, it is necessary to test whether the CRT has a potential to structural reorganization by sports participation and the amount of CRT determines functional reorganization in the ipsilateral M1.

The CRT is a neural pathway that originates primarily in the M1, premotor cortex, and supplementary motor area and projects to the reticular formation in the pons and medulla [18]. The CRT plays a critical role in postural and gait stability. For example, in cats, the activity of CRT neurons in the lateral layer of the reticular formation is modulated in response to gait changes [34]. Previous studies have demonstrated that individuals with LLA who walked with a prosthesis were more unstable than healthy individuals [16]. That is, walking with a prosthesis likely constitutes challenging training for overall balance and gait functions. Therefore, ipsilateral M1 activation would be associated with both the amount of CRT and intensive motor training of whole‐body balance and/or gait functions. As the absence of direct injury to the central nervous system of individuals with LLA, it might be beneficial to investigate the CRT microstructure in healthy individuals to predict the effect of balance training. While our results demonstrate correlations between ipsilateral M1 activation and FC in specific portions of the CRT, we must acknowledge that these findings do not extend to the entire tract. As previous studies using tractometry analysis demonstrated that changes in structural pathway occurred in specific portions of a tract but not in the entire tract [35, 36], the findings did not contradict previous studies. Previous studies have suggested that functional changes are also induced in individuals with upper‐limb amputation [37]. Some studies have pointed out that the pattern of neural plastic changes in individuals with LLA is different from that in upper‐limb amputees [38]. Therefore, our findings are not necessarily applicable to upper‐limb amputees. To clarify this, a comparison of CRT structures between individuals with LLA and upper limb amputees is needed.

Conventional DWI studies often use tract‐based spatial statistics and examine voxel‐wise values such as fractional anisotropy and mean diffusivity [39]. In this tract‐based spatial statistics, the CRT was not evaluated because the white matter atlas did not include the CRT [40, 41]. Few studies have delineated CRT in healthy individuals using tractography [29, 30]. However, these studies did not evaluate the microstructural indices of the CRT (e.g., fractional anisotropy) or the relationship between functions. In addition, voxel‐wise values (e.g., fractional anisotropy) are less conclusive because they are influenced by crossing fibers [42, 43]. Therefore, excluding the influence of crossing fibers is difficult, even though 90% of voxels likely include fibers in several directions [44, 45]. To overcome these limitations, we delineated the CRT and assessed its FC and FD using FBA, which enables the dissociation of fibers within a voxel. Consequently, our findings, which illustrate the structural characteristics in the CRT of individuals with LLA in relation to ipsilateral M1 activation during amputated leg contraction, provide novel evidence of mechanisms in functional reorganization in individuals with LLA.

In several previous studies, structural changes in the CC of individuals with LLA were reported in contrast to those of healthy participants [9, 12, 13]. However, our study did not find any correlation with the CC. Therefore, structural changes in the CC of individuals with LLA may occur independently of ipsilateral M1 activation. One limitation of our study is the absence of a comparison of FC between individuals with LLA and healthy participants matched for age, sex, or sports experience. Previous studies focusing on patients with stroke or spinal cord injury have indicated that changes in alternative pathways may depend on various factors, including time and the nature of the lesions [46, 47]. Therefore, future studies, including healthy controls, are required to investigate structural characteristics by categorizing the site of amputation, years of injury, sports type, as well as the intensity and frequency of the training. We did not perform such a stratified analysis because the sample size in the present study was not sufficiently large. As we excluded amputees with phantom limb pain in the present study because pain might induce cortical reorganization [21], it is also needed to investigate functional–structural relationship in amputees with phantom limb pain.

## 5. Conclusions

FC in the CRT is correlated with ipsilateral M1 activation to the contracted leg during knee contraction in the amputated leg. Thus, structural characteristics in the CRT might stem from ipsilateral M1 activation, which was induced in sport participation. These results demonstrate that functional and structural relationships provide new clinical knowledge on motor rehabilitation and sports participation in LLA. Furthermore, they may contribute to the understanding of neural reorganization in healthy individuals. Specifically, the CRT in humans demonstrates the potential for functional changes in motor control through repetitive motor practice of the lower extremities, although CRT has received less attention compared to CST.

## Funding

This work was funded by JSPS KAKENHI (Grants 18H04082 and 19J21542).

## Conflicts of Interest

The authors declare no conflicts of interest.

## Supporting Information

Additional supporting information can be found online in the Supporting Information section.

## Supporting information


**Supporting Information** Table S1. Values of individual percent signal change during motor task. Figure S1. Scatter plot showing the relationship between fiber cross‐section (FC) and activation in the ipsilateral primary motor cortex, without controlling for age, sex, or years since injury.

## Data Availability

The datasets used and/or analyzed during the current study are available from the corresponding author upon reasonable request.
