# Supplementary material for: Functional–Structural Plasticity Associated With the Duration of Sports Participation in Lower Limb Amputees: A Multimodal Neuroimaging Study
Source: Neural Plast. 2026 Apr 24;2026:8819722. doi: 10.1155/np/8819722 (PMC13107950; doi:10.1155/np/8819722)
Supplement: Supplementary file 1 — Supporting Information Table S1. Values of individual percent signal change during motor task. Figure S1. Scatter plot showing the relationship between fiber cross‐section (FC) and activation in the ipsilateral primary motor cortex, without controlling for age, sex, or years since injury. [file NP-2026-8819722-s001.docx]

Supplementary Materials

As an additional control analysis, percent signal changes (PSCs) in the primary visual cortex (V1) during knee contraction in the amputated leg were extracted. A common coordinate of regions of interest (x = 0, y = −76, z = 10) was used. All other procedures were the same as those used in the control analyses described in the main text. As a result, PSCs in V1 during knee contraction in the amputated leg were not correlated with fiber density (FD) and fiber cross-section (FC), and their combined measure (FDC) (all FWE-corrected ps > 0.05).

Supplementary Table 1　Values of individual percent signal change during motor task

TF: transfemoral amputations; TT: transtibial amputations


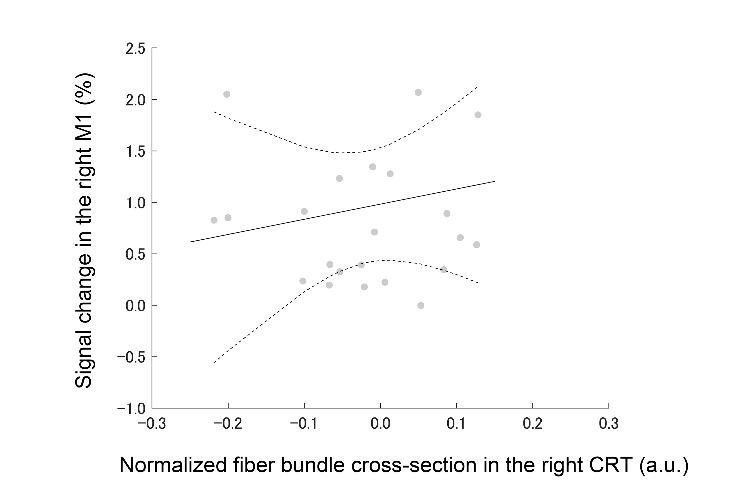


Supplementary Figure 1. Scatter plot showing the relationship between fiber cross-section (FC) and activation in the ipsilateral primary motor cortex, without controlling for age, sex, or years since injury. Please note that individual FC values were calculated relative to the template, which is the default setting in MRtrix3. Therefore, the negative values do not indicate negative CRT metrics; rather, they reflect values lower than the template-based reference. Solid and dotted lines represent the estimated linear regression and 95% confidence interval, respectively. M1: primary motor cortex. CRT: corticoreticular tract.
